# Phosphoproteomics Profiling of Human Skin Fibroblast Cells Reveals Pathways and Proteins Affected by Low Doses of Ionizing Radiation

**DOI:** 10.1371/journal.pone.0014152

**Published:** 2010-11-30

**Authors:** Feng Yang, Katrina M. Waters, John H. Miller, Marina A. Gritsenko, Rui Zhao, Xiuxia Du, Eric A. Livesay, Samuel O. Purvine, Matthew E. Monroe, Yingchun Wang, David G. Camp, Richard D. Smith, David L. Stenoien

**Affiliations:** 1 Pacific Northwest National Laboratory, Richland, Washington, United States of America; 2 Washington State University-Tri-Cities, Richland, Washington, United States of America; 3 Institute of Genetics and Developmental Biology, Chinese Academy of Sciences, Beijing, China; INMI, Italy

## Abstract

**Background:**

High doses of ionizing radiation result in biological damage; however, the precise relationships between long-term health effects, including cancer, and low-dose exposures remain poorly understood and are currently extrapolated using high-dose exposure data. Identifying the signaling pathways and individual proteins affected at the post-translational level by radiation should shed valuable insight into the molecular mechanisms that regulate dose-dependent responses to radiation.

**Principal Findings:**

We have identified 7117 unique phosphopeptides (2566 phosphoproteins) from control and irradiated (2 and 50 cGy) primary human skin fibroblasts 1 h post-exposure. Semi-quantitative label-free analyses were performed to identify phosphopeptides that are apparently altered by radiation exposure. This screen identified phosphorylation sites on proteins with known roles in radiation responses including TP53BP1 as well as previously unidentified radiation-responsive proteins such as the candidate tumor suppressor SASH1. Bioinformatic analyses suggest that low and high doses of radiation affect both overlapping and unique biological processes and suggest a role for MAP kinase and protein kinase A (PKA) signaling in the radiation response as well as differential regulation of p53 networks at low and high doses of radiation.

**Conclusions:**

Our results represent the most comprehensive analysis of the phosphoproteomes of human primary fibroblasts exposed to multiple doses of ionizing radiation published to date and provide a basis for the systems-level identification of biological processes, molecular pathways and individual proteins regulated in a dose dependent manner by ionizing radiation. Further study of these modified proteins and affected networks should help to define the molecular mechanisms that regulate biological responses to radiation at different radiation doses and elucidate the impact of low-dose radiation exposure on human health.

## Introduction

Humans are continuously exposed to low doses of ionizing radiation from both environmental (radon and cosmic rays) and manmade (nuclear power plants and medical procedures) sources, and the health impacts from these exposures are still not well understood[1]. Exposure to these low doses of ionizing radiation could account for some of the frequent cancers that develop as well as other adverse health effects. Numerous studies have documented the effects of high-dose radiation exposure on human health and identified many of the underlying molecular mechanisms that lead to mutations, cancer development and death [2]. A central challenge of radiation research is to understand whether the biological pathways linked to health effects induced by high radiation doses behave in a nonlinear or linear manner at low doses. Implicit in this challenge is the need to understand the underlying mechanisms that govern the overall response of normal tissues exposed to low-dose radiation. In many cases, the effects of low-dose exposure are extrapolated from higher dose studies to assess potential health risks because of the lack of available data on low-dose effects[3]. Emerging evidence, however, suggests that the biological responses to low- and high-dose exposures may be significantly different, as evidenced by altered gene and protein expression profiles[4], [5], altered protein post-translational modifications (PTMs)[6], and findings that cancer risks from low-dose exposure may be overestimated[7]. These investigations show that extrapolation from high-dose experiments may not adequately reflect the low-dose response and point to the need for new studies to explore this issue.

Biological systems are more complex than defined by the genome due in large part to the presence of PTMs that regulate protein activity. Known PTMs on proteins such as histone H2A.X, CHK2, ATM, and p53 undergo very robust changes in response to high doses of radiation compared to changes in protein levels. Phosphorylation, one of the most important and best characterized PTMs[8], is essential in signal transduction, gene regulation, and metabolic control in cells, especially in response to intracellular and extracellular changes and stimuli. Therefore, identification of phosphoproteins, specific phosphorylation sites that regulate protein function, and upstream signaling kinases will provide valuable insight into the molecular mechanisms that regulate the cellular responses to ionizing radiation.

While traditional methods (e.g., immunohistochemistry) typically allow characterization of one phosphoprotein (often only one specific phosphorylation site) at a time, recent advancements in LC-MS technology now enable the broad proteome-wide study of phosphorylation (phosphoproteomics)[9], [10], [11], [12], [13] and enable identification of thousands of phosphorylations sites (and often multiple sites in an individual protein) in a particular proteome. Applying a data analysis pipeline specifically designed to facilitate phosphoproteomics analyses[14], we analyzed alterations in the phosphoproteome present in skin fibroblasts treated with 2 and 50 cGy of ionizing radiation 1 h post-irradiation. A total of 7117 phosphopeptides from control and irradiated primary human skin fibroblasts were identified, which represents a greater than 10-fold improvement on our previously reported phosphoproteomic study [6].

## Results and Discussion

**Figure 1:**
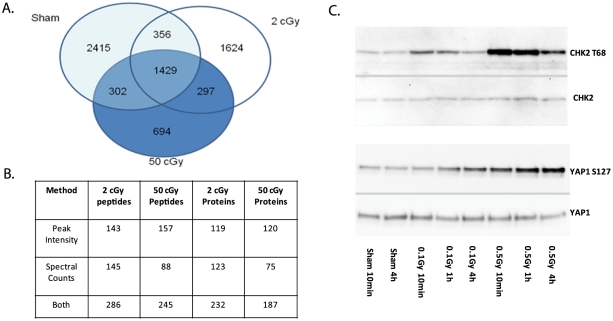
Summary of phosphoproteomic data. A. Venn diagram showing the distribution of phosphopeptides in sham, 2 cGy and 50 cGy treated cells. B. Quantitation of significantly altered peptides using peak intensities and spectral counting methods. C. Validation of YAP1 S127 phosphorylation by Western blotting. YAP1 S127 phosphorylation increases over time at both the lower and higher dose while CHK2 T68 phosphorylation peaks at earlier timepoints. The same samples probed with YAP1 and CHK2 antibodies showed no changes in YAP1 or CHK2 total protein at these timepoints.

To identify molecular targets of low-dose radiation exposure, primary normal human dermal fibroblasts were exposed to 0, 2, or 50 cGy of ionizing radiation and processed 1 h after exposure. Following trypsinization, phosphopeptides were enriched using IMAC and subjected to LC-MS/MS. A total of 7117 total unique phosphopeptides with a False Discovery Rate (FDR) of ≤0.5%[14] were identified and distributed across each condition as shown in **Figure 1A**, and the complete list of phosphopeptides identified from four technical replicates is shown in **Table S1**.

Statistical methods used in comparative quantitation of peptides detected in label-free MS experiments can be used to facilitate comparison across different conditions and are typically based on comparison of the peak intensities of the MS parent ion or the number of MS/MS spectral counts from each peptide[15]. While these methods are semi-quantitative in nature, they are frequently used for comparative analyses of peptide and protein abundance differences. In the case of whole proteins, multiple peptides from the parent protein can be used to gain confidence in the quantitation. With phosphorylation site analysis, there is usually only one available peptide so that there is the potential for a larger margin for error in the statistical analysis. With data from four technical replicates available, we expect that semi-quantitative evaluation can reveal apparent phosphorylation changes caused by the low-dose radiation, which as a proof-of-principle, will provide the rationale for future, more comprehensive and accurate quantitative experiments by employing several biological replicates, static isotopic labeling such as iTRAQ and validation experiments with antibodies, and site-directed mutagenesis.

With this caveat in mind we employed the two semi-quantitative methods based on spectral counting and peak intensities to identify phosphopeptides that are apparently altered in the 2- and 50-cGy samples relative to the sham irradiated control (**Figure 1B**). Application of the spectral count based G-test revealed 145 2-cGy and 88 50-cGy phosphopeptides with abundance significantly different from control at 95% confidence. Application of the T-test to the peptide ion intensity data identified 143 2-cGy and 157 50-cGy phosphopeptides, respectively, showing 2.5-fold and higher changes in abundance at 95% confidence. Given the different nature of these tests, these two methods provide largely non-overlapping phosphopeptides. Combining the data from the two methods identified 286 2-cGy and 245 50-cGy phosphopeptides corresponding to 233 2-cGy and 187 50-cGy affected proteins. The top phosphopeptides showing the greatest fold change for peak intensities and spectral counts are shown in **Tables 1** and **2**, and the complete list of significantly changed phosphopeptides can be found in **Tables S2** and **S3**.

A notable example showing overlap between the spectral count and peak intensity data is the SSRP1 phosphopeptide containing phospho-serine 444 (S444) for which we identified an 8- and 6.5-fold increase in the phosphorylation by spectral counting and a 6.5- and a 4.9-fold increase in peak intensities at 2 and 50 cGy, respectively (**Tables 1** and **2**). SSRP1 along with Spt16 comprises the FACT complex, which acts as a histone chaperone to temporarily displace histones during transcription[16], [17]. It has also been recently shown that FACT catalyzes the exchange of H2AX at sites of DNA damage[18] and regulates homologous recombination during DNA repair[19] suggesting that the radiation-induced phosphorylation could play a role in regulating this process.

**Table 1:** Top phosphopeptides identified by peak intensities.

Gene	Peptide	T-test: 2 cGy:C	T-test: 50 cGy:C	2 cGy/C	50 cGy/C	Site	PotentialKinase
SASH1	R.TCS*FGGFDLTNR.S	**0.0034**	ND	24.7	ND	S407	AKT
NEXN	K.AEIKEMLAS*DDEEDVSSKVEK.A	**0.0007**	**0.0024**	13.7	21.5	S80	CK II
C11orf58	R.S*ASPDDDLGSSNWEAADLGNEER.K	**0.0012**	**0.0036**	21.4	14.6	S15	None
BYSL	R.MPQDGS*DDEDEEWPTLEK.A	**0.0108**	**0.2031**	21.1	22.6	S98	CK II
MAP1B	R.DVMS*DETNNEETESPSQEFVNITK.Y	**0.0450**	**0.0140**	6.8	20.2	S1144	CK II
PXN	K.TGSSS*PPGGPPKPGSQLDSMLGSLQSDLNK.L	**0.0016**	**0.0144**	14.4	14.8	S288	Erk1
GFPT1	R.VDS*TTCLFPVEEK.A	**0.0239**	**0.0064**	14.2	3.5	S243	AMPK
AHSG	K.CDSSPDS*AEDVRK.V	**0.0428**	**0.0115**	12.0	4.1	S138	DNA PK
PLEC1	R.TS*SEDNLYLAVLR.A	**0.0092**	**0.0001**	12.0	10.4	S20	PKA
CDC42EP1	K.NAIS*LPQLNQAAYDSLVVGK.L	**0.0006**	**0.0019**	8.4	11.3	S121	None
ATP2B1	R.IEDS*EPHIPLIDDTDAEDDAPTK.R	**<.0000**	**<.0000**	8.6	10.8	S1193	CK II
CFL1	K.LGGS*AVISLEGKPL.-	**0.0006**	**0.0072**	10.8	10.3	S155	None
LEO1	K.KYVIS*DEEEEDDD.-	**0.0211**	**0.0024**	6.2	9.6	S658	CK II
TBC1D4	R.SLTSS*LENIFSR.G	**0.0026**	**0.0016**	9.0	7.3	S570	PKC
SR-A1	R.FDIYDPFHPTDEAYS*PPPAPEQK.Y	**0.0015**	**0.0027**	6.3	8.8	S239	Erk1
ZC3H13	R.NTEESS*SPVRK.E	**0.0379**	0.2075	8.8	7.2	S111	None
MAP4	K.TEVALAKDMES*PTKLDVTLAK.D	**0.0004**	**0.0012**	8.6	6.0	S280	Cdk5
SEPT2	K.IYHLPDAES*DEDEDFKEQTR.L	**0.0032**	**<.0000**	7.9	8.5	S218	CK II
ARHGEF6	K.S*TAALEEDAQILK.V	**0.0199**	**0.0177**	6.3	8.3	S649	PKC
ATP2B1	R.IEDS*EPHIPLIDDTDAEDDAPTKR.N	**0.0043**	**0.0151**	8.2	6.6	S1193	None
MATR3	R.RDS*FDDRGPSLNPVLDYDHGSR.S	**0.0001**	**0.0013**	6.1	8.2	S188	CLK2
HDAC1	R.MLPHAPGVQMQAIPEDAIPEES*GDEDEDDPDKR.I	**0.0368**	**0.0177**	5.4	8.2	S393	CK II
PLEC1	R.T*SSEDNLYLAVLR.A	**0.0021**	**<.0000**	8.1	7.0	T19	PKA
ITGA4	R.RDS*WSYINSK.S	**0.0334**	**0.0068**	7.8	4.4	S1027	PKA
CEP170	R.QGS*FTIEKPSPNIPIELIPHINK.Q	**0.0105**	**0.0014**	2.8	7.7	S838	CAMK2G
USP8	R.SYS*SPDITQAIQEEEK.R	**0.1368**	**0.0092**	7.8	7.2	S718	AKT
EIF5B	K.WDGS*EEDEDNSK.K	**0.0245**	**0.0009**	7.7	4.9	S164	CK II
TP53BP1	K.MVIQGPSS*PQGEAMVTDVLEDQKEGR.S	**0.0176**	**0.0436**	6.2	7.2	S1114	Erk1
IFI16	K.VSEEQTQPPS*PAGAGMSTAMGR.S	**0.0179**	**0.0252**	0.15	0.24	S153	ERK1
WRNIP1	K.RPAAAAAAGSAS*PR.S	0.1212	**0.020**	0.36	0.096	S151	None

The T-test was used to identify significantly affected phosphopeptides based on relative peak intensities. The complete list can be found in supplementary Table 2.

**Table 2:** Top phosphopeptides identified by spectral counts.

Gene	peptide	Control	2 cGy	50 cGy	2 cGY GTest	50 cGY GTest	Site	Potential Kinase
PRKCDBP	APEPLGPADQSELGPEQLEAEVGES*S*DEEPVESR	0	0	30	ND	**37.856**	S165, S166	CK II
PGRMC1	GDQPAASGDS*DDDEPPPLPR	16	0	2	**19.938**	**9.381**	S56	CK II
PRKAR2A	VADAKGDS*ES*EEDEDLEVPVPSR	38	11	11	**16.764**	**11.450**	S77, S79	CK II
NOP58	HIKEEPLS*EEEPCTSTAIASPEK	0	13	1	**15.094**	0.523	S502	ATM
FAM21B	SPMFPALGEASS*DDDLFQSAK	0	2	12	**1.450**	**13.783**	S1092	CK II
GPR124	ALPAAAEDGS*PVFGEGPPSLK	11	0	1	**13.074**	**7.183**		CDK5
OSBP	MLAES*DES*GDEESVSQTDKTELQNTLR	0	7	11	**7.340**	**12.479**	S190, S193	CK II
SSRP1	EGMNPSYDEYADS*DEDQHDAYLER	2	16	13	**11.324**	**8.912**	S444	CK II
RALGPS2	CHS*LGYNFIHK	0	10	2	**11.181**	1.456	S343	PKC
PLEKHA5	TNS*MQQLEQWIK	0	10	5	**11.181**	**4.876**	S410	PKA
SRRM1	TRHS*PT*PQQSNR	11	3	0	**4.890**	**10.706**	S414, T416	CDK5
NIPBL	AITSLLGGGS*PK	0	9	0	**9.893**	ND	S2658	CDK5
BAZ1B	LAEDEGDS*EPEAVGQSR	8	0	1	**9.038**	**4.372**	S1468	CK II
AHNAK	AS*LGSLEGEAEAEASSPK	6	22	19	**8.948**	**7.904**	S5749	PKC
TJAP1	GS*PEEELPLPAFEK	0	8	4	**8.615**	3.680	S300	PKA
JUN	LQALKEEPQTVPEMPGET*PPLS*PIDMESQER	0	8	1	**8.615**	0.523	T239, S243	GSK3
MAP1A	WLAES*PVGLPPEEEDKLTR	0	8	6	**8.615**	**6.102**	S1776	ERK1
SORBS3	LCDDGPQLPTS*PR	1	11	3	**8.603**	0.957	S530	CDC2
LMNA	ASSHSSQTQGGGS*VTK	9	2		**4.700**	**8.463**	S414	PKC
LARP1	ESPRPLQLPGAEGPAIS*DGEEGGGEPGAGGGAAGAAGAGR	7	0	5	**7.716**	**4.876**	S90	CK II
IGF2BP2	ISYIPDEEVSSPS*PPQR	7	0	0	**7.716**	**6.262**	S164	CDK5
SGPP1	NS*LTGEEGQLAR	7	0	0	**7.716**	**6.262**	S112	PKA
IWS1	AAVLS*DS*EDEEKASAK	8	2	0	3.750	**7.356**	S398, S400	CK II
MGMT	GAGATSGS*PPAGRN	8	2	0	3.750	**7.356**	S201	CDC2
SNW1	GPPS*PPAPVMHS*PSR	8	2	0	3.750	**7.356**	S224, S232	ERK1, CDC2
IRS2	TYS*LTTPAR	0	5	7	**4.870**	**7.350**	S577	AKT
AKAP12	VLSKPPEGVVSEVEMLSS*QER	0	1	7	0.520	**7.350**	S505	ATM
IRF2BP1	AGGAS*PAASSTAQPPTQHR	7	8	0	0.030	**6.262**	S453	none
PFKP	GRS*FAGNLNTYK	7	6	0	0.117	**6.262**	S386	AKT
CHD3	METEADAPS*PAPSLGER	7	1	0	**4.665**	**6.262**	S1660	GSK3

The G-test was used to identify significantly affected phosphopeptides based on the number of observed spectra. The complete list can be found in Supplementary Table 3.

To validate our proteomics data, Western blots were performed using available phosphorylation-specific antibodies. While most of the identified phosphorylation sites have not been extensively studied, and therefore, few phosphorylation specific antibodies are available, Yes Associated Protein 1 (YAP1) S127 is known to be phosphorylated on the site that serves to regulate its apoptotic and transcriptional activity [20]. Using the phosphorylation-specific antibody, we confirmed by Western blot that the S127 site undergoes increased phosphorylation after radiation treatment in agreement with the mass spectrometry data showing a 2.6-fold increase following exposure to radiation (**Figure 1C**). In the case of YAP1, phosphorylation increases over time (up to 4 h) especially at low doses. In contrast, Chk2 T68, which was not detected in the current screen but is also phosphorylated at low doses, peaks at early time points indicating that different phosphorylation sites have different temporal and dose kinetics.

### Protein kinases affected by radiation

To determine if specific kinases were activated by exposure of cells to 2 or 50 cGy ionizing radiation, we performed a kinase motif analysis using the Scansite website (http://scansite.mit.edu [21]). Shown in **Tables 1** and **2** are the top potential kinases for each identified phosphopeptide. Casein kinase II (CK II) consensus sites were the most prominent and several studies have linked CK II as a key regulator of ionizing radiation responses [22], [23]. Several sites predicted to be phosphorylated by DNA damage regulated kinases, ATM, and DNA protein kinase (DNA PK) were also identified, and most of the CK II sites could also be phosphorylated by DNA PK due to the close similarities of these motifs. Other kinases for which consensus motifs were identified include AKT, ERK, PKC, and PKA.

### Biological pathways affected by radiation exposure

**Figure 2:**
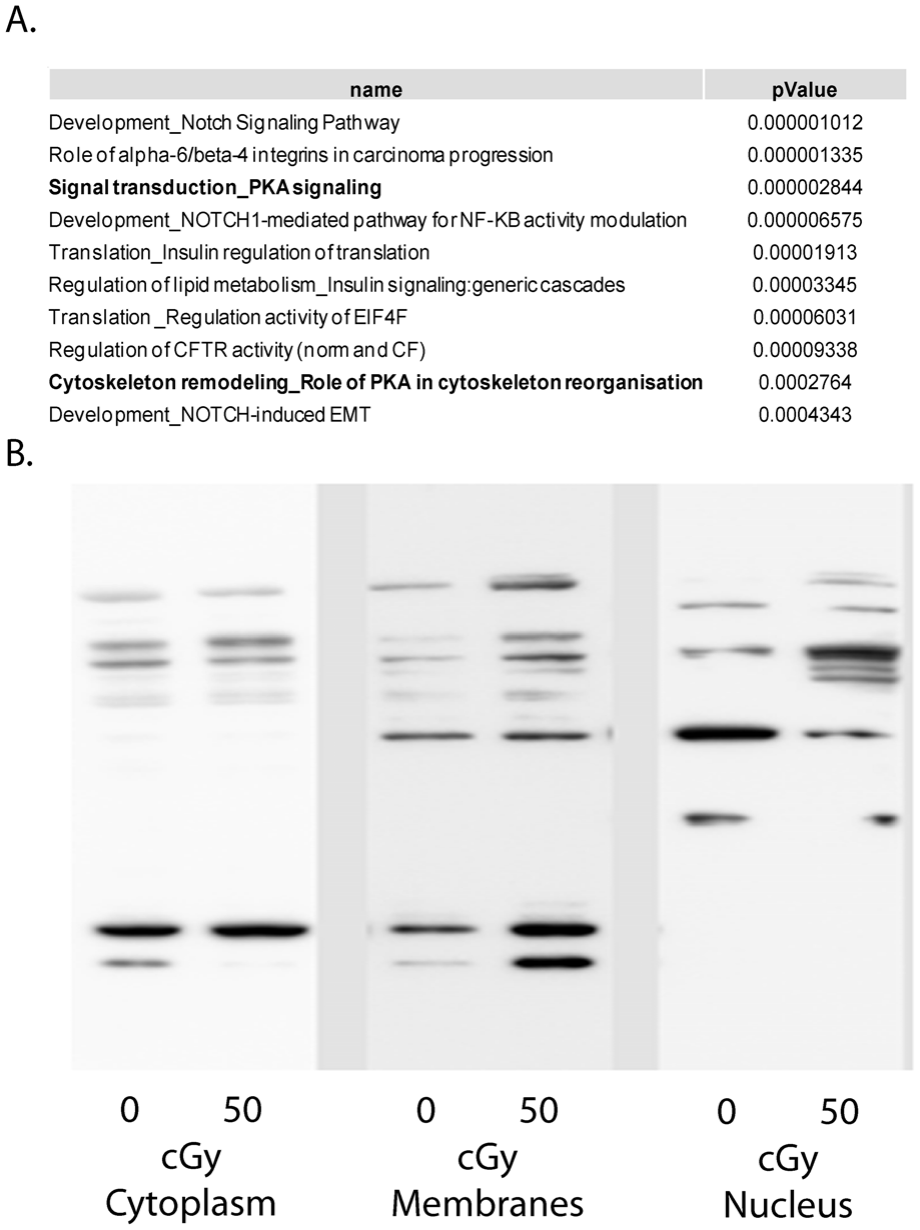
Implication of PKA signaling in radiation response. A. Top molecular pathways affected by radiation identified by MetaCore. B. Western Blotting with PKA motif antibody demonstrates that PKA substrates are differentially phosphorylated following radiation exposure in different subcellular compartments. NHDF cells were exposed to 0 or 50 cGY of ionizing radiation and subcellular fractionation was performed 1 h post-radiation.

Comparison of the 2-cGy and 50-cGy datasets shows that there are 121 overlapping phosphopeptides, 166 unique 2-cGy phosphopeptides, and 123 unique 50-cGy phosphopeptides. Rolled up to the protein level, there were 118 overlapping proteins, 113 unique 2-cGy proteins and 64 unique 50-cGy proteins. To identify specific biological pathways affected by radiation, bioinformatics analyses were performed using the MetaCore software from GeneGo, Inc. (St Joseph, MI). Among the top radiation-affected pathways (both 2- and 50-cGy datasets) identified by MetaCore was the PKA signaling pathway (**Figure 2**). Specific components of this pathway included the PKA regulatory subunit (PRKAR2A); the PKA interacting proteins AKAP2, AKAP11 and AKAP12, which regulate PKA activity by anchoring this kinase to specific intracellular domains[24]; and 3-phosphoinositide dependent protein kinase-1 (PDK1), which phosphorylates and activates the catalytic domain of PKA[25] (**Table 3**). All of these proteins were significantly affected at the phosphorylation level in the 2- and 50-cGY treated cells with the exception of AKAP11, which was not significantly altered at 2 cGy.

**Table 3:** Kinases and regulators affected by radiation.

Gene	Peptide	T-test: 2 cGy	T-test: 50 cGy	Change2 cGy	Change 50 cGy
ABL1	K.GQGESDPLDHEPAVS*PLLPR.K	**0.0113**	**0.0008**	2.6	2.9
AKAP11	R.SVS*PTFLNPSDENLK.T	0.1771	**0.0046**	2.7	2.0
MAPK1	R.VADPDHDHTGFLTEY*VATR.W	**0.0009**	**0.0031**	2.8	2.5
PDPK1	R.ANS*FVGTAQYVSPELLTEK.S	**0.0043**	**0.0132**	3.5	3.2
PRKAB2	R.DLSSS*PPGPYGQEMYAFR.S	**0.0009**	**0.0106**	3.6	2.7
		G-Test: 2 cGy	G-Test: 50 cGy	Spectra: 0:2 cGy	Spectra: 0:50 cGy
PRKAR2A	VADAKGDS*ES*EEDEDLEVPVPSR	**16.76**	**11.45**	38∶11	38∶11
AKAP12	VLSKPPEGVVSEVEMLSS*QER	0.52	**7.35**	0∶1	0∶7
AKAP2	TNGHS*PSQPR	**6.73**	**4.94**	11∶ 2	11∶ 2
MAP3K11	NVFEVGPGDS*PTFPR	**5.13**	0.23	5∶0	5∶3
MAP4K4	RDS*PLQGSGQQNSQAGQR	0.78	**4.37**	8∶5	8∶1
PRKCDBP	APEPLGPADQSELGPEQLEAEVGES*S*DEEPVESR	0	**37.86**	0∶0	0∶35
PRKD1	RLS*NVSLTGVSTIR	1.28	**4.13**	5∶2	5∶0
EGFR	ELVEPLT*PSGEAPNQALLR	**5.13**	**4.13**	5∶0	5∶0

Shown is a partial list of the kinases and regulatory proteins affected by 2 and 50 cGY of radiation. The top group of peptides were identified using the peak intensity methods and T-test for quantification. The fold change for each peptide relative to control is shown. The bottom group of peptides were identified based on spectral counts using the G-test to assess significance. In both cases, those peptides showing significant change from control values are shown in bold.

PKA phosphorylates a general consensus motif of KRXXpS/pT or RRXpS/pT[26], [27] although there are many cases in the literature showing that PKA phosphorylates other sites as well. Using an antibody recognizing the RRXpS motif (Phospho-PKA Substrate #100G7E, Cell Signaling Technology), we were able to demonstrate that a number of PKA substrates are affected by radiation exposure (**Figure 2**). These changes were not readily evident when whole cell lysates were analyzed (data not shown), but when Western blots were performed on subcellular fractions, a number of PKA substrates were observed to be altered in radiation-exposed cells compared to sham-irradiated cells. We observed both increased and decreased phosphorylation of these substrates indicating that the radiation treatment does not lead to a general increase in PKA activity, but that the effects are targeted to specific PKA substrates in different subcellular compartments. Given the role of AKAPs in regulating PKA localization and activity, it is plausible to speculate that the altered PKA phosphorylation patterns could be attributed to the differential AKAP phosphorylation observed.

To identify PKA substrates within our dataset, we searched the significantly altered phosphopeptides for PKA consensus phosphorylation motifs using Scansite and identified 49 phosphopeptides that contain a potential PKA phosphorylation motif. The 2-cGy dataset contained 36 PKA phosphopeptides while the 50-cGy dataset contained 42 PKA phosphopeptides. In agreement with our Western blot data we observed both increased (42 phosphopeptides) and decreased (7 phosphopeptides) PKA-dependent phosphorylation. The complete list of PKA substrates is presented in **Table S4**.

To determine if other protein kinase signaling pathways were affected by radiation, we analyzed our datasets for the presence of protein kinase pathway members that are phosphorylated or dephosphorylated after radiation treatments, which could account for their altered activity. Shown in **Table 3** is a partial list of kinases and kinase regulatory proteins affected by radiation treatment. Of interest is the finding that several kinase pathways with known links to radiation effects are directly affected at the phosphorylation level by radiation. Proteins showing altered phosphorylation include several components of the MAP kinase signaling pathway including MAPK1 (ERK2), MAP3K11, and MAP4K4. ERK phosphorylation is known to increase following exposure to low and high doses of ionizing radiation and may play a role in regulating the adaptive response of low-dose radiation to high challenging doses [28], [29]. Activation of ERK2 signaling is associated with dual phosphorylation of both T184 and Y186, while here we observe only the single phosphorylation at Y186. Also of interest is the finding that oxysterol-binding protein 1 (OSBP) is phosphorylated on several sites (S190, S193, S238 and S240) in both the 2- and 50-cGy samples. OSBP functions as a cholesterol sensing scaffolding protein that may regulate ERK1/2 activity by binding to and regulating the tyrosine and threonine phosphatases that act on the T184 and Y186 sites on ERK2[30]. Recently, functional studies of OSBP S240 phosphorylation by protein kinase D (PKD) demonstrated the involvement of this site in regulating the localization of OSBP to the Golgi apparatus[31]. PKD S205 phosphorylation was also significantly increased in the 50-cGy treated cells (PRKD1 gene product; **Table 3**). This phosphorylation site on PKD regulates apoptosis signal-regulating kinase 1 (ASK1) association and c-Jun N-terminal kinase (JNK) signaling cascades under conditions of oxidative stress[32].

Other important signaling proteins of note include Epidermal Growth Factor Receptor (T369), c-Abl (S569), and Protein Kinase C Delta binding protein (PRKCDBP; S165; S166), which undergoes a large change in phosphorylation at 50 cGy only. EGFR regulates radiation responses through activation of AKT and phosphatidylinositide 3-kinase (PI3K). Recent evidence suggests that EGFR may have a radioprotective effect that is mediated through its translocation to the nucleus[33], where it acts to repair DNA damage[33]. T669 is a major ERK-dependent phosphorylation site on EGFR following EGF stimulation and is involved in receptor internalization[34], suggesting a link between the observed radiation-induced phosphorylation and EGFR involvement in DNA damage repair.

### DNA damage repair proteins

A number of proteins associated with radiation responses and DNA damage repair were identified in our screen. Prominent among these is p53-binding protein 1 (53BP1), which translocates to intranuclear foci containing gamma-H2AX following treatments with radiation doses as low as 1 cGy[35], where it plays an important role in DNA damage recognition[36]. We identified two 2-cGy sites (S1114 and S1462) and three 50-cGy sites (S831, S1114, and S1317) that exhibited an increase in phosphorylation at these radiation doses (**Tables S2** and **S3**). Interestingly, the majority of these phosphorylation sites are present within the minimal domain required for foci localization[37], suggesting that the phosphorylation could play a role in regulating 53BP1 localization.

### Biological pathways affected by low doses of radiation

A critical question in low-dose radiation research is to determine if a linear no threshold (LNT) model is valid for assessing human health risks of low-dose radiation exposure. A number of studies have called into question the validity of the LNT model by showing that many radiation effects involving DNA damage repair, transcriptional activation, and apoptosis as well as animal epidemiological data show both qualitative and quantitative differences at low versus high doses of radiation[38].

Many of the phosphopeptides we identified were present in both the 2- and 50-cGy datasets, suggesting that many of the signaling mechanisms are conserved at low- and high-dose exposures. The KEGG pathways that were most affected by radiation exposure, shown in **Table 4**, are comparable at the two doses in terms of the numbers of proteins involved in each pathway. The top signaling pathways affected by radiation include insulin signaling through PRKA and MAPK signaling, both mentioned above. Interestingly, only the spliceosome pathway showed a dose effect, involving many more members at the high dose than the low dose. SNW1, also known as SKI interacting protein, is required for SKI oncoprotein transforming activity and has been shown to release the growth suppressive activity of the retinoblastoma tumor suppressor[39], [40].

**Table 4:** KEGG pathways affected by radiation.

KEGG pathway	2 cGy		50 cGy	
	Count	Proteins	Count	Proteins
Insulin signaling pathway	8	MAPK1, PDPK1, IRS2, EIF4EBP1, PRKAR2A, TSC1, PRKAB2, TSC2	6	PDPK1, IRS2, PRKAR2A, EIF4EBP1, TSC1, PRKAB2
Pathways in cancer	7	EGFR, MAPK1, CCDC6, HDAC1, RALBP1, JUN, ABL1	5	EGFR, HDAC1, PML, LOC652671, ABL1
MAPK signaling pathway	6	EGFR, MAPK1, JUN, RRAS, STMN1, MAP3K11	6	EGFR, MAP4K4, NF1, RRAS, NFATC4, STMN1
mTOR signaling pathway	6	EIF4B, MAPK1, PDPK1, EIF4EBP1, TSC1, TSC2	4	EIF4B, PDPK1, EIF4EBP1, TSC1
Tight junction	6	EPB41L2, RAB3B, TJP1, MAGI1, RRAS, TJAP1	4	EPB41L2, MAGI1, RRAS, TJP2
Adherens junction	6	EGFR, MAPK1, TJP1, BAIAP2, LMO7, VCL	3	EGFR, LMO7, CTNND1
Endocytosis	5	EGFR, DAB2, RABEP1, SH3KBP1, IQSEC1	4	EGFR, USP8, SH3KBP1, IQSEC1
Spliceosome	1	SF3B2	6	SFRS4, SFRS9, SNW1, SFRS1, PRPF38B, SF3B2

The DAVID web portal was used to calculate statistical enrichment of KEGG pathways.

**Figure 3:**
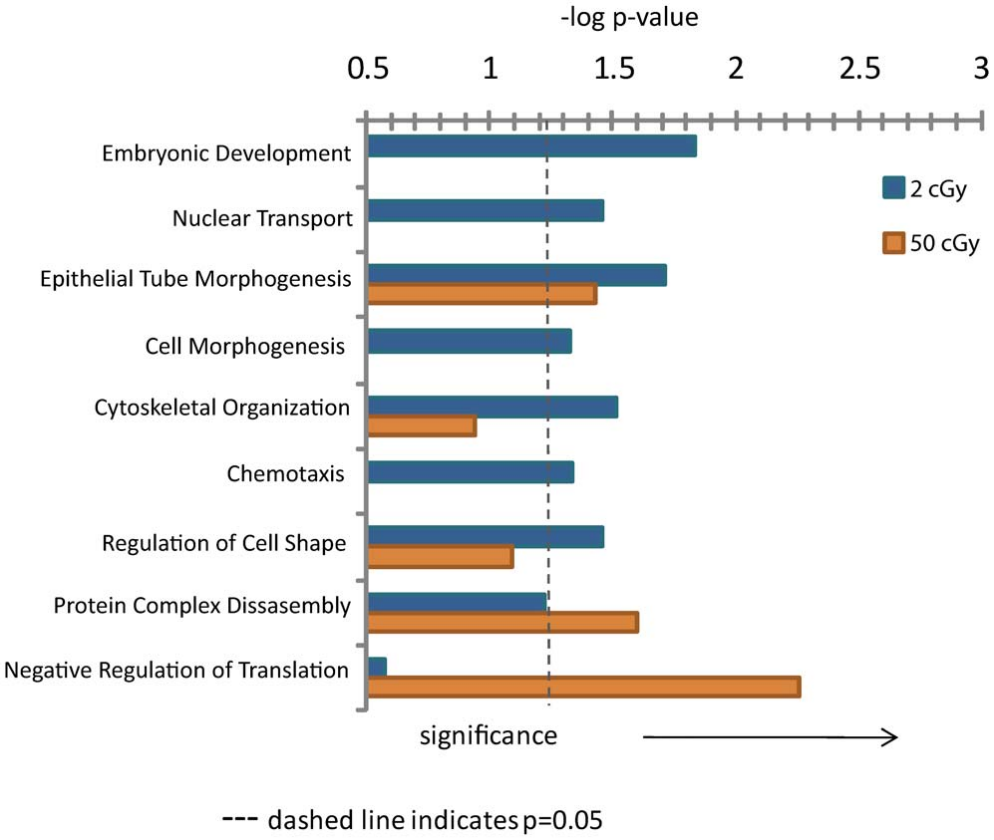
Biological processes affected by radiation. Shown are the log p values for each dose group, with the dashed line indicating statistical significance (p = 0.05) identified using the DAVID web portal.

We also observed a large number of 2-cGy phosphopeptides that were not significantly affected in 50-cGy affected cells, suggesting that there may be signaling mechanisms that are unique to low-dose exposures. Using statistical enrichment analysis, we identified those biological processes that were affected by either 2- or 50-cGy exposures (**Figure 3**). Multiple proteins involved in embryonic development, nuclear transport, cell morphogenesis, and chemotaxis were phosphorylated by 2-cGy exposure, while proteins involved in negative regulation of translation were significantly phosphorylated by 50-cGy exposure. Most notably responsive to the 2-cGy dose group was the chemotaxis process, which contains RALBP1 and TSC2. RALBP1 aids in the clearance of glutathione-conjugated electrophilic compounds, such as biproducts of oxidative stress, and loss of this protein is associated with sensitivity to radiation[41]. The TSC-mTOR pathway (also shown in **Table 4**) is a key regulator of innate immune homeostasis, whose dysregulation has been shown to be a contributor to tumor development[42], [43].

**Figure 4:**
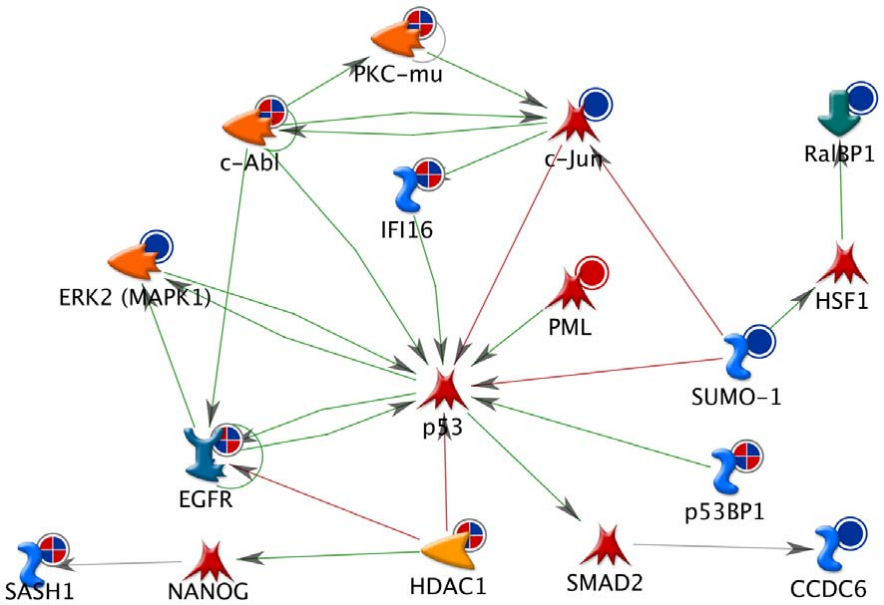
p53 regulatory network affected by radiation exposure. The blue dots adjacent to specific proteins indicate phosphorylation change by low dose, red dots indicate phosphorylation change by high dose, and red/blue hatched dots indicate change by both dose groups. Green edges (lines) between proteins indicate activation of the protein at the head of the arrow, and red edges indicate inactivation.

A closer look at the p53 regulatory network for the KEGG cancer pathway is shown in **Figure 4**. Blue dots indicate a phosphorylation change with a low dose of radiation and red dots indicate a phosphorylation change with a high dose of radiation. Several proteins have red/blue hatched dots, which indicate that their phosphorylation state was affected by both low and high doses of radiation. Interestingly, at low doses of radiation there are two proteins, c-Jun and SUMO-1, that are uniquely affected by low dose and act as repressors of p53. The c-Jun protein has a double phosphorylation on T239 and S243 found only in the 2-cGy sample. The S243 phosphorylation is known to regulate c-Jun protein stability [44] and possibly affect the c-Jun role in proapoptotic signaling following radiation exposure[45]. At higher doses of radiation, there is phosphorylation of PML, which is a known p53 activator[46]. IFI16, which plays a role in p53-dependent DNA repair and transcriptional pathways and also regulates the production and secretion of multiple chemokines and cytokines driving the initial steps in the inflammatory process, is dephosphorylated following 2- and 50-cGy exposures[47]. Through this network, it is possible that p53 is differentially regulated leading to dose-dependent changes in p53 transcriptional activity [48] and different p53 dependent phenotypic effects, such as adaptive responses at low doses [49] and apoptotic responses at higher doses[50].

Perhaps the strongest evidence for unique low-dose-dependent signaling is the finding that some phosphopeptides undergo very large changes only in the 2-cGy treated cells making it unlikely that these changes are due to random chance. A good example of this is SASH1, a candidate tumor suppressor[51], [52], [53], for which we observed an ∼25-fold increase in phosphorylation at S407 only at the 2-cGy dose (**Table 1** **)**. In addition to S407, we also observed significant changes in SASH1 phosphorylation at S837 and S839 (**Table S2)** at both 2- and 50-cGy doses indicating that this protein has multiple dose-dependent phosphorylation sites. Other phosphoproteins uniquely affected by 2-cGy treatment and showing many-fold changes in phosphorylation include c-jun as mentioned above; Nipbl, which is mutated in Cornelia de Lange syndrome and reportedly affects DNA damage sensitivity in these patients[54]; and Vinexin (SORBS3), which interacts with and is phosphorylated by ERK1/2, mediating the effects of EGF signaling on anchorage-dependent growth[55]. Further analysis of these proteins and their PTMs should help to provide mechanistic insight into the physiological implications of low dose radiation exposure.

## Materials and Methods

### Reagents

All reagents were obtained from Sigma-Aldrich (St. Louis, MO) unless otherwise noted below. Antibodies used in Western blots (YAP1, YAP1 pS127, Chk2, Chk2 pT68, and Phospho-PKA Substrate #100G7E) were obtained from Cell Signaling Technology (Beverly, MA).

### Cell Culture

Primary normal human dermal fibroblast (NHDF) cells were obtained from Lonza (Rockland, ME) and cultured in Fibroblast Growth Media-2 (FGM-2; Lonza). Cells were grown in 150-mm plates to confluence for 2 days to induce growth arrest and irradiated with 0, 2-, or 50-cGy low-LET radiation generated using a Pantek XRAD 320 irradiator (GE Inspection Technologies, General Electric Co., Hurth, Germany). Cell fractionation experiments were performed using the Subcellular Protein Fractionation Kit (Thermo Fisher Scientific, Rockford, IL).

### Protein Digestion and Cleanup

1 h post irradiation, cell lysates were prepared by direct addition of freshly made 8 M urea in 50 mM ammonium bicarbonate (pH 8.0) containing a 1X concentration of phosphatase inhibitor cocktail (Thermo Fisher Scientific, Rockford, IL) followed by scraping cells and shearing DNA using an 18 gauge needle. After incubation on ice for 30 minutes, insoluble matter was removed by centrifugation at 16,000×g for 20 minutes.

### Protein Digestion and Methyl Esterification

Samples were diluted 2-fold with 50 mM NH_4_HCO_3_ (pH 7.8) and digested with sequencing grade modified trypsin (Promega, Madison, WI) at a trypsin to protein ratio of 1∶100 (w/w) at room temperature for 6 h with constant shaking at 300 rpm in Thermomixer R (Eppendorf, Hauppauge, NY), and then were further diluted 4-fold, followed by an additional 16 h at room temperature after treatment with a second aliquot of trypsin at the same trypsin-to-protein ratio. While trypsin is only partially active in 4 M urea, performing the first digestion at this concentration enables digestion of the denatured proteins into larger fragments that are completely digested using trypsin in 1 M urea. We find this two-step digestion provides superior digestion of difficult to digest proteins. To prevent potential carbamylation of proteins in urea [56], we used freshly made urea buffer and performed digestions at room temperature.

Samples were acidified with Trifluoroacetic acid (TFA) (0.5% final concentration TFA) to interrupt digestion. The tryptic digests were ultracentrifuged (166,000×g for 30 min at 4°C) to remove nucleic acids prior to SPE desalting[57]. Each desalted sample (500 µg) was completely dried using a Speed-Vac SC 250 Express (Thermo Savant, Holbrook, NY). For peptide methyl esterification, 40 µl of thionyl chloride was added drop-wise to 1 ml anhydrous methanol with constant stirring. After ∼5 min, the mixture was cooled and added at ∼100 µL/100 µg ratio to the dried peptide, sonicated for 10 min at room temperature, and vortexed for another 5 min at room temperature. After incubation at room temperature for 1 h, the methyl esterified peptides were completely dried again using a Speed-Vac concentrator and kept at -80°C until time for further use.

### Phosphopeptide Enrichment and Capillary HPLC-MS/MS Analysis

Methyl esterified peptides were subjected to Fe^3+^ immobilized metal affinity chromatography (IMAC) to enrich phosphopeptides. A custom-packed IMAC Macrotrap cartridge (3 mm i.d. ×8 mm length) (Michrom BioResources, Inc., Auburn, CA) was employed for phosphopeptide enrichment. Briefly, using a syringe pump, the IMAC cartridge was activated with 500 µL of 100 mM FeCl_3_ at 25 µL/min, the excess metal ions were removed with 250 µL of 0.01% acetic acid (HOAc) at 50 µL/min, and the column was equilibrated with 500.0 µL of wash buffer (1∶1∶l of 0.01% HOAc/Acetonitrile/Methanol) at 50 µL/min. Methyl esterified peptides from each sample condition were resuspended in 250 µL of wash buffer, and the pH was adjusted using 10% ammonium hydroxide to ∼3.5. The sample was then loaded onto the IMAC cartridge at 4 µL/min and washed with 100 µL of wash buffer at 25 µL/min and with 600 µL of wash buffer at 50 µL/min. The IMAC column was then re-equilibrated with 500 µL of 0.01% HOAc at 50 µL/min before the phosphopeptide elution using 250 µL of 250 mM Na_2_HPO_4_ pH∼8.0 at 10 µL/min. The eluate was immediately acidified with TFA to pH ∼3.5-4.

After IMAC enrichment, aliquots (1/12 of the IMAC eluate) were analyzed on a ThermoElectron LTQ-Orbitrap (Waltham, MA) coupled to an automated dual-column phosphoproteome nano-HPLC platform assembled in-house[58]. The LC gradient (*A* = 100 mM HOAc in H_2_O, *B* = 70% acetonitrile/100 mM HOAc in H_2_O) was 0-70% B for ∼180 min. Full-scan mass spectra were acquired over an m/z range of 300 to 1575 and either MS/MS for the top 10 abundant species or multi-stage activation (MSA) for the top 6 abundant species for a given MS scan was used for peptide fragmentation. Survey spectra were acquired with a resolution of 60,000 in the Orbitrap while acquiring tandem mass spectra in the LTQ part of the instrument. For MSA, during fragmentation, the neutral loss species resulting from phosphate loss and a combination of water and phosphate loss at 97.97, 115.97, 48.99, 57.99, 32.66, or 38.66 m/z below the precursor ion were activated in turn for 30 ms each[59]. For each sample, 4 technical replicates of MS analyses were performed.

### Database search and peptide identification

Phosphopeptides were identified from MS/MS spectra using SEQUEST (Sequest Cluster version 27 revision 12 from Bioworks 3.2, ThermoElectron Corp., Waltham, MA) by searching against the Human IPI database (version 3.20, 61,225 protein sequences, www.ebi.ac.uk/IPI, European Bioinformatics Institute, Cambridge, UK). The search parameters were: (1) Fully tryptic peptide termini (allowing ≤2 missed cleavages) (amino- and carboxy-termini were considered tryptic termini). (2) Dynamic modifications: an addition of 79.9663 Da to serine, threonine, and tyrosine residues (phosphorylation). (3) Static modifications: an addition of 14.0157 Da to aspartic acid, glutamic acid, and the carboxy-terminus (methyl esterification). (4) Precursor ion mass tolerance: ±0.05 Da. (5) Fragment ion mass tolerance: ±0.5 Da (*m/z*). (6) Maximum number of the same amino acid that can be dynamically modified in a phosphopeptide: 3. The false discovery rate (FDR) of phosphopeptide identification was controlled at ≤ 0.5% using our in-house developed software[14]. This program also measured the probability of correct phosphorylation site localization in each identified peptide by calculating the AScore for each phosphorylation site using Gygi's approach[60], which is shown in **Table S1**.

### Statistical analysis for phosphopeptide changes with radiation exposure

We use both spectral count and peak area methods to evaluate the changes of phosphopeptides. The total spectral counts for each identified phosphopeptide in each radiation condition and their ratios were used to semi-quantitatively estimate their abundance in each condition[61]. The G-test was used to assess significance of spectral count data at the 95% confidence level[62]. We also used the peak areas of phosphopeptides identified in each of four MS technical replicates to evaluate the phosphopeptide abundance changes. Cross-correlation analyses of each replicate (supplementary **Figure S1**) were performed and generated scores ranging from 0.79 to 0.93, where 100% correlation between replicates would yield a score of 1. Briefly, the peak area for each phosphopeptide was extracted from the MS data applying the MASIC program developed in-house[63] and used to assess phosphopeptide abundance. To obtain significant changed phosphopeptides under control and each radiation condition, the T-test was performed for the phosphopeptides identified from each condition across four MS technical replicates, and p<0.05 was used to filter the non-significantly changed proteins. All significantly changed phosphopeptides were combined for the final list using either spectral count or peak area methods.

### Bioinformatics Analyses

Phosphorylated protein lists were used to determine biological pathways affected by radiation exposure. The MetaCore software (GeneGo Inc, St Joseph, MI) was used to identify common molecular pathways at both doses and to create regulatory networks for selected pathways. The DAVID web portal[64], [65] was used to calculate statistical enrichment of KEGG pathways and Gene Ontology biological processes for each dose group separately. Process groups were considered significant with at least 5 protein members and p<0.05. Kinase motif analysis was performed using the Scansite web tool (http://scansite.mit.edu [21]).

## Supporting Information

Figure S1 Cross correlation analysis of technical replicates. Phosphopeptide correlation plots (Log 2 Peak intensities) within 4 LC-MS/MS technical replicates for each condition are shown. The red line corresponds to Y = X. The data points along the X or Y axis are unique to one technical replicate. The correlation values are shown diagonally across the correlation plot for the 2 replicates compared.
(2.85 MB EPS)

Table S1 Shown is the complete list of identified phosphopeptides.
(6.92 MB XLS)

Table S2 Significantly altered phosphopeptides based on peak intensities.
(0.08 MB XLS)

Table S3 Significantly alterered phosphopeptides based on spectral counts. The G-test was used to identify significantly affected phosphopeptides based on the number of observed spectra. NS  =  not significant.
(0.06 MB XLS)

Table S4 Potential PKA substrates identified using SCANSITE.
(0.21 MB XLS)
